# A Domain-General Developmental “Do-GooD” Network Model of Prosocial Cognition in Adolescence: A Systematic Review

**DOI:** 10.3389/fnbeh.2022.815811

**Published:** 2022-03-08

**Authors:** Benjamin S. Sipes, Tony T. Yang, Kendall C. Parks, Namasvi Jariwala, Olga Tymofiyeva

**Affiliations:** ^1^Department of Radiology and Biomedical Imaging, University of California, San Francisco, San Francisco, CA, United States; ^2^Department of Psychiatry and Behavioral Sciences, The Langley Porter Psychiatric Institute, Division of Child and Adolescent Psychiatry, Weill Institute for Neurosciences, University of California, San Francisco, San Francisco, CA, United States

**Keywords:** prosocial, adolescence, networks, theory of mind, MRI, domain general cognitive processes

## Abstract

Adolescence is a period of substantial neural and social development, and prosocial decisions are beneficial to personal well-being, the well-being of others, and the functioning of society. Advances in network neuroscience call for a systematic synthesis and reappraisal of prosocial neural correlates during adolescent development. In this systematic review, we aim to outline the progress made in this field, identify the similarities between study results, and propose a model for prosocial cognition in adolescents to young adults. A total of 25 articles were included in this review. After reviewing and synthesizing the literature, we propose a DOmain-General Developmental “Do-GooD” network model of prosocial cognition that aligns with the reviewed literature, accounts for development, and combines elements of the value-based decision-making model with distinct value contributions from the default mode network, salience network, and control network. We offer predictions to test the “Do-GooD” model and propose new future directions for studying prosocial behavior and its development during adolescence, which in turn may lead to improving education and the development of better health interventions for adolescents.

## Introduction

Adolescence is a period of substantial neural and social development. Prosocial behavior, defined as voluntary behavior intended to benefit another individual (Eisenberg et al., 2007), is a key element to human cooperation in society, it relates to health benefits for the giver (Inagaki and Eisenberger, 2016; Moieni et al., 2019), and it is thought to be causally related to improved well-being [for reviews, see Helliwell et al. (2017) and Aknin et al. (2019)]. While prosocial behavior is multi-dimensional with many possible incentives, altruistic prosociality is dominated by motivations to benefit others without obvious self-benefit (Batson, 1991), and thus it may reflect intrinsically motivated helping behavior. Consolidating magnetic resonance imaging (MRI) research on altruistic prosocial behavior could elucidate prosocial decision-making mechanisms significant to adolescence, which could inform the development of interventions aimed at improving prosocial development in youth.

Prosocial behavior in childhood predicts adulthood prosociality (Eisenberg et al., 2001), and prosocial development during early adolescence appears highly influenced by the social-cultural environment (House et al., 2013). Both childhood prosocial behavior and social-cultural environment highlight adolescence as a critical period for healthy prosocial development. Competencies related to prosocial behavior are known to develop during adolescence, such as decision-making (Mann et al., 1989) and interpersonal relationships (Smetana et al., 2006). The adolescent brain also undergoes substantial structural remodeling in the gray matter, white matter, and functional activity related to social-cognition [see for review Blakemore (2012)].

Neural mechanisms of prosocial behavior and cognition are under active investigation. MRI neuroimaging has an advantage over other imaging modalities by providing excellent spatial resolution and whole-brain coverage. Functional MRI (fMRI) in particular is able to identify regions with greater blood oxygen level dependence (BOLD) signal during prosocial tasks compared to control tasks. Bellucci et al. (2020) conducted a recent meta-analysis on this neuroimaging literature, showing that brain regions associated with prosociality included the ventromedial prefrontal cortex (VMPFC), left dorsolateral prefrontal cortex (DLFPC), middle cingulate cortex (MCC), and the posterior cingulate cortex (PCC). The authors also noted that prosocial brain regions had significant overlap with mentalizing (or Theory of Mind) regions in the VMPFC and PCC, as well as with an empathy region in the MCC (Bellucci et al., 2020).

To our knowledge, no models have been developed to explicitly describe prosocial decision-making during development. Research on Theory of Mind (ToM), which is the ability to attribute mental states to self and others (Goldman, 2012), has been often suggested to underly prosocial behavior through greater involvement in other-oriented brain regions that facilitate empathy and develop during adolescence (Cassidy et al., 2003; Decety and Jackson, 2004; Carter and Huettel, 2013; Slaughter et al., 2015; Meinhardt-Injac et al., 2020). Value-based decision-making as a general model for decision-making in the brain has also been speculated to describe altruism (Brosch and Sander, 2013). One neuroeconomic model proposed by Declerck et al. (2013) uses a value-based decision-making framework to characterize prosocial behavior in adulthood, proposing that different brain systems are responsible for different prosocial motivations: that altruism primarily involves social brain regions, while selfish incentives (e.g., money or reputation) drive prosocial behavior through cognitive control brain regions (Declerck et al., 2013). Yet none of these models synthesize findings into a general understanding of prosocial neurodevelopment.

Recent advances in human neuroscience research emphasize the importance of brain networks for describing general cognitive processes (Bassett and Sporns, 2017). Domain-general networks such as the medial frontoparietal “default-mode” network (DMN), the midcingulo-insular “salience” network (SN), and the lateral frontoparietal “control” network (CN) are identified in resting state brain activations and during task-evoked cognition (Uddin et al., 2019), which may suggest their role in facilitating cognitive processes such as prosocial behavior. Each network’s functionality is varied, but a few broad patterns have emerged. The DMN is likely involved in generating predictions (Dohmatob et al., 2017), consolidating social information (Meyer et al., 2019), and theory of mind (Hyatt et al., 2015). The SN is strongly connected to deep-brain structures involved in bodily sensation (Kleckner et al., 2017), empathy (Fan et al., 2011), and evaluating fairness (Gabay et al., 2014). The CN is generally implicated in self-control (Berkman et al., 2017), but it also is involved in understanding social norms (Hackel et al., 2020) and evaluating moral preferences (Crockett et al., 2017). Moreover, accumulating evidence has highlighted significant development both within and between the DMN, SN, and CN during adolescence (Uddin et al., 2011; Ryali et al., 2016). Yet to current scientific knowledge, no accounts of prosocial behavior in terms of such domain-general networks exist.

This present work systematically reviews the literature on prosocial cognition spanning adolescent development with three goals. Our first aim is to summarize the common findings on the neural correlates of adolescent prosocial behavior grouped by domain-general networks. That review informs our second aim in which we propose a **DO**main-**G**eneral **D**evelopmental “Do-GooD” model of prosocial cognition that synthesizes many of the previously described models into a domain-general network framework that can explain the reviewed results and some of their heterogeneity. Our third and final aim is to offer new predictions based on the “Do-GooD” model to guide advances in the field of adolescent prosocial neural development.

## Methods

We reviewed and synthesized the literature on MRI neural correlates of prosocial development in typically developing adolescents from three electronic databases as described by the following sections. We conducted this systematic review following PRISMA recommendations (Moher et al., 2009).

### Search Strategy

We conducted our search on 16th April 2021 in three databases: PubMed, PsychINFO, and Web of Science. An updated search was conducted on 8th November 2021.

The following search terms with Boolean operators queried the databases’ titles and abstracts: [(adolescen* OR “young adults”) AND (fMRI OR DTI OR MRI OR “magnetic resonance”) AND (prosocial OR pro-social OR altruis* OR coop* OR trust OR “trust game” OR recipro*) AND (behavior* OR behaviour*)]. To ensure search results from recently published work, we searched Google Scholar using the terms [adolescent AND prosocial AND mri] published since the year 2020, and evaluated the first twenty most relevant results for eligibility.

### Screening, Eligibility and Data Extraction

We removed duplicate results, then screened abstracts and methods sections for all articles. The following criteria were considered a reason for exclusion: not a full research paper, not peer-reviewed, the publication was not in English, assessed a strictly adult (over 22) or childhood (under 10) population, no MRI imaging, animal studies, case studies, no prosocial measures relating to MRI. Our age criterion was based on past work and reviews on adolescence (Steinberg, 2008; Van Hoorn et al., 2019), and we included studies with subjects ages 10 to 22 years. We excluded studies where the mean subject age was below 10- and above 22-years-old. Full-text articles were then assessed for eligibility. Studies upon closer inspection that did not have a clear relationship between prosocial decisions and neural correlates were further excluded from the analysis. Since we focused on the altruistic dimension of prosocial behavior, we excluded studies with tasks where adolescents’ optimal strategy to maximize self-payoff was to be prosocial, which does not reflect altruistic choices. Lastly, we excluded atypical populations because the aim was to review healthy prosocial decision-making and development.

The remaining studies were reviewed, and their data were extracted. Extracted information included the following: the first author’s name, year published, a general summary of study aims, study sample sizes, population ages, MRI modality, any tasks performed by the subjects during/after the MRI, the study’s measure of prosociality, the resulting neural correlates in their experiment, and any correlations/effect-sizes corresponding to those neural correlates.

## Results

**FIGURE 1:**
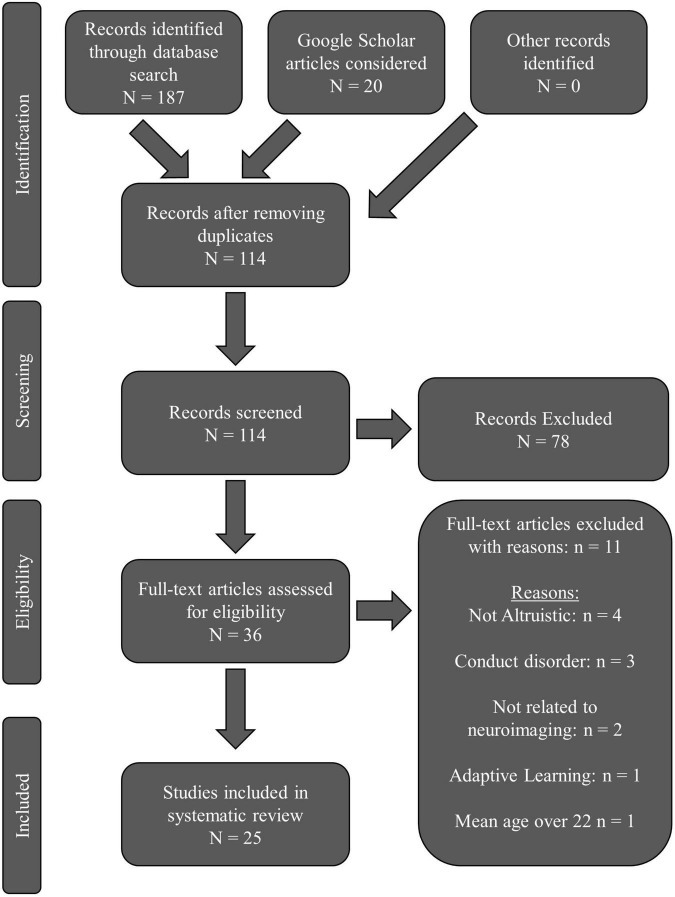
PRISMA flow chart for the systematic review literature search.

The PRISMA flow chart is presented in Figure 1. Our database search revealed 187 total articles: PubMed returned 60 results; PsychINFO returned 35 results; Web of Science returned 92 results. Twenty articles were assessed from Google Scholar. After removing all duplicates, 114 articles remained. Thirty-six articles remained after initial screening for eligibility. Eleven full-text articles were excluded: four were not altruistic; three focused on conduct disorder; two did not relate prosocial findings to neuroimaging; one focused on adaptive learning; one had young adults with a mean age over 22-years-old. Twenty-five total articles were reviewed.

The 25 included studies are summarized in Table 1. In the following, we describe the types of methodologies used to study prosocial behavior. We then describe neural correlates related to prosociality in sections focusing on reward and valuation regions, the default mode network, the salience network, the control network, and visual/somatosensory networks. We close each section with a summary of the main findings.

**TABLE 1:** This table summarizes all results from the reviewed studies.

Author (Year)	Total N	Age range(s)	Prosocial measure	MRI measure	Aspect of prosociality	Analytic approach	Contrast	Regions implicated
Brandner et al., 2021	142 (88 female)	8 to 19	Donation observation	Charity or Self Yield Task (task-based fMRI)	Prosocial Choice	Region of Interest	Mother Gain > No Gain & Father Gain > No Gain & Stranger Gain > No Gain	Nucleus accumbens (mother/father) > stranger
						Region of Interest Correlation	Mother Gain > No Gain & Father Gain > No Gain	nucleus accumbens (positive association with pleasure) Mother Gain (*r* = 0.25), Father Gain (*r* = 0.28)
						Region of Interest	Father Gain > No Gain & Stranger Gain > No Gain	VMPFC (Father) > VMPFC (Stranger)
						Region(s) of Interest Correlation	Mother Gain > No Gain & Father Gain > No Gain & Stranger Gain > No Gain Correlation of nucleus accumbens and VMPFC	Mother Gain (*r* = 0.61) Father Gain (*r* = 0.66) Stranger Gain (*r* = 0.47)
Do et al., 2019	51 (28 female)	8 to 16	Donation behavior	Allocation Task (task-based fMRI)	Prosocial Choice	Whole-Brain Contrast	Costly Giving > Non-costly Giving	B-pSTS, L-Prec, L-inferior temporal gyrus, L-IFG, L-DLPFC, R-DMPFC
					Prosocial Choice/Frequency	Whole-Brain Regression	Costly Giving > Costly Reward	dACC (negative association)
Do and Telzer, 2019	51 (28 female)	8 to 16	Donation behavior	Allocation Task (task-based fMRI)	Prosocial Choice	Region of Interest	Give Out-Group > Give In-Group	FC between VS and R-pSTS
Duell et al., 2021	97 (51% female)	11 to 14	Donation behavior	Charity time donation (task-based fMRI)	Decision-Making	Whole-Brain Contrast	Decisions Post > Pre-observation	B-insula, B-inferior temporal gyrus, L-middle occipital gyrus, R-dACC, R-FFG, R-postcentral gyrus, R-cuneus
				hormone interaction with task activation	Decision-Making	Whole-Brain Regression	Post > Pre-obs * cortisol * Testosterone	B-OFC, PCC, B-cerebellum, L-TPJ, L-insula, L-DLPFC, L-precentral gyrus, R-caudate, R-MTG, R-superior orbital gyrus
Ferschmann et al., 2019	169 (92 female)	12 to 26	SDQ (prosocial)	Cortical Thinning	Frequency	Whole-Brain Regression	N/A	L-DLPFC, R-pMTG, R-IFG, R-mPFC, R-IPS, R-TPJ, R-dACC
Masten et al., 2010	20 (10 female)	12 to 13	Prosocial writing (post-task)	Cyberball Observation (task-based fMRI)	Frequency	Whole-Brain Regression	Exclusion > Inclusion	R-AI (*r* = 0.71), R-PCC (*r* = -0.68), R-Prec (*r* = -0.69)
Moor et al., 2012	53 (13 female)	10 to 12 14 to 16 19 to 21	% forgiving offers	Dictator Game (task-based fMRI)	Frequency	Whole-Brain Regression	Excluders > Includers	dACC (positive association)
					Decision-Making	Whole-Brain Contrast	Excluders > Includers (19-21)	dACC, L-TP, R-insula
Güroğlu et al., 2014	10	μ = 20.7	Donation behavior	Dictator Game (task-based fMRI)	Prosocial Choice	Whole-Brain Contrast	Prosocial Inequity > Equity	VMPFC, VS, R-insula
	28 (17 female)				Frequency	Whole-Brain Regression	Decision-making	Prec, VMPFC, R-DLPFC (positive associations)
Lemmers-Jansen et al., 2018	47 (22 female)	16 to 27 (μ = 21)	Social Mindfulness	SoMi Task (task-based fMRI)	Decision-Making	Whole-Brain Contrast	Decision-Making Prosocial > Control	B-DMPFC, B-middle frontal gyrus, B-IFG, B-TPJ, L-ACC, L-IPL, R-MCC, R-PCC, R-Prec
					Prosocial Choice	Conjunction Analysis	Prosocial Decisions	L-IPL, L-Prec, R-DLPFC, R-IFG, R-TPJ, R-MTG, R-cuneus
Okada et al., 2019	271 (129 female)	10 to 13	SDQ (prosocial)	RS-FC in ACC	Frequency	Region of Interest	RS-FC with ACC correlating with SDQ	B-MCC, B-PCC, R-precentral gyrus (all positive associations)
				MRS on ACC	Frequency	Region of Interest	metabolites with SDQ	GABA (ρ = -0.15)
Overgaauw et al., 2014	37 (23 female)	12 to 19	Donation behavior	Dictator Game (task-based fMRI)	Frequency	Whole-Brain Regression	Negative > Positive social scenes correlation with giving	R-IPL (*r* = -0.35)
Sakai et al., 2017	45 (0 female)	15 to 16	Donation behavior	AlAn Game (task-based fMRI)	Decision-Making	Whole-Brain Contrast	Decision > Control	mPFC, ACC, caudate, thalamus, VTA, B-insula, B-IFG, R-DLPFC, R-SPL, R-IPL, R-TPJ, R-postcentral gyrus
Schreuders et al., 2018	27 (12 female)	μ = 21.25	Donation behavior	Dictator Game (task-based fMRI)	Frequency	Whole-Brain Regression	Decision-making Friend > Disliked Peer	SMA, L-lingual gyrus, L-precentral gyrus, R-insula, R-DLPFC, R-calcarine gyrus (all negative associations)
					Prosocial Choice/Frequency	Whole-Brain Regression	% prosocial Friend > Disliked peer	SMA (*r* = -0.6), R-insula (*r* = -0.62)
					Prosocial Choice	Whole-Brain Contrast	Prosocial Friends > Disliked Peers	B-TPJ/IPL, L-putamen, R-IFG
					Prosocial Choice	Whole-Brain Contrast	Prosocial Friends > Unknown Peers	B-TPJ/IPL, L-SPL, L-Prec
Schreuders et al., 2019	50 (21 female)	μ = 14.6	Donation behavior	Dictator Game (task-based fMRI)	Prosocial Choice	Whole-Brain Contrast	Prosocial Friends > Disliked Peers	B-SPL, R-postcentral/precentral gyri, R-MTG, R-insula, R-TPJ, R-middle occipital gyrus, R-putamen
					Prosocial Choice	Whole-Brain Contrast	Prosocial Friends > Unknown Peers	B-SPL, B-IPL, L-middle occipital gyrus, L-precentral gyrus
Spaans et al., 2020	160 (84 females)	11 to 21	Donation observation	Charity or Self Yield Task (task-based fMRI)	Prosocial Choice	Whole-Brain Contrast	Charity-Gain > Both-No-Gain	B-TPJ, VMPFC, L-mPFC, L-DLPFC, R-Prec
			Donation behavior (post-task)	Charity or Self Yield Task (task-based fMRI)	Prosocial Choice	Region of Interest	Charity-Gain > Both-No-Gain	nucleus accumbens (positive association)
Tashjian et al., 2018	20 (7 female)	13 to 15	Donation behavior (post-task)	Prosocial, Social, or Neutral scene observation (task-based fMRI)	Frequency	Conjunction based Regression Analysis	Prosocial > Neutral&Social scenes	B-TPJ (positive association)
Telzer et al., 2011	25 (13 female)	μ = 20.2	Donation behavior	Family Assistance Task (task-based fMRI)	Prosocial Choice	Whole-Brain Contrast	Costly Donation > Non-costly Reward	B-IPL, L-DPLFC, R-DMPFC
					Prosocial Choice	Whole-Brain Regression	Costly Donation > Non-costly Reward WBR family obligation	B-pSTS, R-TPJ, R-ACC, L-DLPFC
					Prosocial Choice	Region of Interest	Costly Donation > Non-costly Reward FC	FC between VS and L-VLPFC, L-DMPFC, R-mPFC
Telzer et al., 2013	32 (18 female)	15 to 16	Donation behavior	Family Assistance Task (task-based fMRI)	Prosocial Choice	Whole-Brain Contrast	Costly Donation > Non-costly Reward	dACC, cuneus, ventral midbrain, L-insula
					Prosocial Choice	Whole-Brain Regression	Costly Donation > Non-costly Reward WBR family obligation	B-VS
Tousignant et al., 2018	20 adolescents (10 female) & 20 adults (10 female)	μ adoles = 14.25 μ adult = 24.25	Cyberball inclusion	Cyberball Observation (task-based fMRI)	Frequency	Regression on significant whole-brain regions	Observation Exclusion > Inclusion correlated with behavior in young adults	insula (*r* = 0.46), amygdala (*r* = 0.47)
				Cyberball Play (task-based fMRI)	Decision-Making	Whole-Brain Contrast	Play Exclusion > Inclusion	PCC, B-TPJ, B-TP (extending to insula and VMPFC), R-mPFC, R-DMPFC, R-DLPFC, R-lateral temporal cortex, R-caudate
				Cyberball Play (task-based fMRI)	Decision-Making	Whole-Brain Contrast	Play Exclusion > Inclusion adults > adolescence	R-TPJ, R-DMPFC/mPFC, R-fusiform face area
van den Bos et al., 2009	22 (11 female)	18 to 22	Reciprocity	Trust Game (task-based fMRI)	Prosocial Choice	Whole-Brain Contrast	Reciprocate > Defect	B-visual cortex
					Frequency	Whole-Brain Regression	Defect > Reciprocate% reciprocate	dACC, B-insula, L-Prec, R-TPJ, R-thalamus (positive associations); R-VS (negative association)
van den Bos et al., 2011	62 (30 female)	12 to 14 15 to 17 18 to 22	Reciprocity	Trust Game (task-based fMRI)	Prosocial Choice	Whole-Brain Contrast	Reciprocate > Defect	B-visual cortex
					Prosocial Choice	Whole-Brain Contrast and *post hoc* correlation	Reciprocate > Control (age correlation)	mPFC (r = -0.56)
					Frequency	Whole-Brain Regression	Defect > Reciprocate WBR%reciprocate	dACC, B-insula (positive association)
					Decision-Making	Whole-Brain Contrast	Decision-making > Control	B-dorsal striatum, L-Prec, R-SPL, R-DLPFC, R-DMPFC, R-ACC
					Decision-Making	Whole-Brain Contrast	Decision-making (older > younger)	L-TPJ, R-DLPFC
van der Meulen et al., 2016	23 (all female)	18 to 19	Cyberball inclusion	Cyberball play (task-based fMRI)	Prosocial Choice	Whole-Brain Contrast	Prosocial > Control	B-TPJ, B-cuneus, B-insula, L-nucleus accumbens, R-IFG, R-superior temporal gyrus
Van Hoorn et al., 2016	61 (31 female)	12 to 13 15 to 16	Donation behavior	Public Goods Game (task-based fMRI)	Decision-Making	Whole-Brain Contrast	Decision-making Observation > Alone	B-TPJ/STS, B-Prec, B-DMPFC
					Decision-Making	Region of Interest based on significant whole-brain activation	Age*Condition interaction	DMPFC, L-STS
Will et al., 2016 *	43 (17 female)	μ = 14.1	Donation behavior	Dictator Game (task-based fMRI)	Frequency	Whole-Brain Regression	% Forgiveness Excluders > Includers	DMPFC
Telzer et al., 2010 and Will et al., 2018*	47 (18 female)	μ = 14	Donation behavior	Dictator Game (task-based fMRI)	Decision-Making	Whole-Brain Contrast	Decision-making Costly equity > (Non-costly equity + non-costly giving)	B-striatum, B-pre-SMA, B-VMPFC, L-pSTS/TPJ, L-calcarine gyrus, L-MTG, L-SMA, L-IFG, L-ACC, R-PCC, R-fusiform gyrus
					Decision-Making	Whole-Brain Contrast	Decision-making Non-costly giving > (Non-costly equity + Costly equity)	B-middle occipital gyrus

B- (bilateral-); L- (left-lateralized-); R- (right-lateralized-); Prec (precuneus) mPFC (medial prefrontal cortex); DMPFC (dorsal medial prefrontal cortex); DLPFC (dorsolateral prefrontal cortex); VLPFC (ventrolateral prefrontal cortex); IFG (inferior frontal gyrus); ACC (anterior cingulate cortex); dACC (dorsal ACC); MCC (middle cingulate cortex); PCC (posterior cingulate cortex); SPL (superior parietal lobule); IPL (inferior parietal lobule); TPJ (temporoparietal junction); STS (superior temporal sulcus); pSTS (posterior STS); STG (superior temporal gyrus); MTG (middle temporal gyrus); pMTG (posterior MTG); TP (temporal pole); VS (ventral striatum); VTA (ventral tegmental area); GABA (gamma-aminobutyric acid); WBR (whole-brain regression); AlAn (Altruism Antisocial Game); SoMi (Socially Mindful Task); MRS (magnM,etic resonance spectroscopy); *Will et al. (2016, 2018) had overlapping (non-independent) adolescent samples.

### Methodological Strategies to Study Prosociality in Adolescents

Among the 25 included articles, seven reported prosocial neural correlates related to behaviors that were not imaged directly (see Table 1, column “Prosocial Measure”). Two of these used the self-report Strengths and Difficulties Questionnaire’s (SDQ) prosocial subscale related to cortical thickness changes (Ferschmann et al., 2019) and resting state functional connectivity (Okada et al., 2019). The other five studies used a prosocial behavioral task after neuroimaging and related prior neural activations to subsequent prosociality (Masten et al., 2010; Overgaauw et al., 2014; Tashjian et al., 2018; Tousignant et al., 2018; Spaans et al., 2020).

Nineteen studies reported prosocial neural correlates concurrently with a prosocial decision-making task. Seven of these used the Dictator Game (Moor et al., 2012; Güroğlu et al., 2014; Will et al., 2016, 2018; Schreuders et al., 2018, 2019; Duell et al., 2021), and five used close variations on the Dictator Game, including either the Allocation Game (Do and Telzer, 2019; Do et al., 2019), the Family Assistance Task (Telzer et al., 2011, 2013), or the Charity of Self Yield Task (Spaans et al., 2020; Brandner et al., 2021). The seven remaining studies used various games that also involved resource distribution: the Socially Mindful task (Lemmers-Jansen et al., 2018), the Altruism Antisocial Game (Sakai et al., 2017), the Trust Game (van den Bos et al., 2009, 2011), the Public Goods Game (Van Hoorn et al., 2016), and Cyberball (van der Meulen et al., 2016; Tousignant et al., 2018).

In addition to the methodological differences described above, the reviewed studies focused on three different aspects of the decision-making process (see Table 1, column “Aspect of Prosociality”). Eight studies compared (pro)social decision-making in general (i.e., the deliberation phase) to neutral/non-prosocial control conditions (hereafter, “prosocial decision-making”) (van den Bos et al., 2011; Moor et al., 2012; Van Hoorn et al., 2016; Sakai et al., 2017; Lemmers-Jansen et al., 2018; Tousignant et al., 2018; Will et al., 2018; Duell et al., 2021). Eleven studies contrasted prosocial decision-making trials based on the actual prosocial *choice* such that decision-making with prosocial outcomes were compared to decision-making with non-prosocial outcomes (hereafter, “prosocial choices”) (Telzer et al., 2011, 2013; Güroğlu et al., 2014; Lemmers-Jansen et al., 2018; Schreuders et al., 2018, 2019; Do and Telzer, 2019; Do et al., 2019; Spaans et al., 2020; van der Meulen et al., 2016; Brandner et al., 2021). Twelve studies analyzed neural activation during prosocial decision-making that correlated with the *frequency* of making prosocial choices, and therefore accounts for between-person differences (hereafter, “giving frequency” or “behavior frequency”) (van den Bos et al., 2009, 2011; Masten et al., 2010; Moor et al., 2012; Güroğlu et al., 2014; Overgaauw et al., 2014; Will et al., 2016; Schreuders et al., 2018; Tashjian et al., 2018; Tousignant et al., 2018; Ferschmann et al., 2019; Okada et al., 2019).

### Reward and Valuation Regions

The reviewed studies often identified the striatum as related to prosociality in adolescents. Studies that analyzed prosocial decision-making found activation in the bilateral striatum (Will et al., 2018) including the dorsal striatum (van den Bos et al., 2011) and the caudate (Sakai et al., 2017; Tousignant et al., 2018; Duell et al., 2021). Prosocial choices across adolescence frequently implicated the ventral striatum (VS) (van den Bos et al., 2009; Telzer et al., 2013; Güroğlu et al., 2014) including the nucleus accumbens (Spaans et al., 2020; van der Meulen et al., 2016; Brandner et al., 2021), with greater VS activation when decisions were prosocial. The VS also had significant functional connectivity relating to prosocial choices in other regions, including the posterior superior temporal sulcus (pSTS) adolescents behaved equitably with outgroups (Do and Telzer, 2019), and the left ventrolateral prefrontal cortex (VLPFC), left dorsomedial prefrontal cortex (DMPFC), and right medial prefrontal cortex (mPFC) in young adults making costly donations (Telzer et al., 2011). The putamen also activated in adolescents (Schreuders et al., 2019) and young adults (Schreuders et al., 2018) while making prosocial choices toward friends more than to disliked peers. The midbrain was also identified during both decision-making (Sakai et al., 2017) and prosocial choices (Telzer et al., 2013). Lastly, the VMPFC was identified in youth during prosocial decision-making (Güroğlu et al., 2014; Tousignant et al., 2018; Will et al., 2018; Duell et al., 2021), its activation during decision-making interacted with pubertal hormone changes (Duell et al., 2021), and its activation was greater for prosocial choices (Güroğlu et al., 2014; Spaans et al., 2020; Brandner et al., 2021). One study additionally found a correlation between nucleus accumbens and VMPFC activation during prosocial choices (Brandner et al., 2021).

#### Summary

In the reviewed literature, brain regions involved in reward and value processing were related to prosocial behavior. Both the striatum and VMPFC were involved in prosocial decision-making, and they had greater activation when youth made prosocial choices. Some striatal subregions may be more involved in (pro)social decision-making generally, while others were involved specifically in making prosocial choices.

### Default Mode Network Regions

The reviewed studies identified default mode network (DMN) regions as related to prosociality in adolescence. Gray matter cortical thinning rate was greater in the mPFC, temporoparietal junction (TPJ), inferior frontal gyrus (IFG), and medial temporal gyrus (MTG) for adolescents who scored as highly prosocial on the SDQ compared to those who scored as low prosocial (Ferschmann et al., 2019). While only one study showed changes in the brain’s structure, many others showed DMN activation and developmental differences during prosocial tasks.

The mPFC, and specifically the DMPFC, both activated during prosocial decision-making, and most frequently activation was right-lateralized (Telzer et al., 2011; van den Bos et al., 2011; Tousignant et al., 2018; Do et al., 2019) or bilateral (Van Hoorn et al., 2016; Sakai et al., 2017; Lemmers-Jansen et al., 2018). Four studies also found prosocial choices related to D/MPFC activation that was left-lateralized (Spaans et al., 2020), right-lateralized (Telzer et al., 2011; Do et al., 2019), or bilateral (Will et al., 2016). Some studies suggest that adolescent development relates to activation changes during prosocial tasks, where young adults had greater activation in the right DMPFC (Telzer et al., 2011; Tousignant et al., 2018) but decreasing activation in the mPFC (van den Bos et al., 2011).

The PCC and precuneus had activation that predicted prosocial frequency occurring after a non-prosocial fMRI task (Masten et al., 2010). The PCC and precuneus were identified bilaterally active during both prosocial decision-making and prosocial choices in adolescents (Van Hoorn et al., 2016; Tousignant et al., 2018; Will et al., 2018; Do et al., 2019; Spaans et al., 2020) and young adults (van den Bos et al., 2009; Güroğlu et al., 2014; Lemmers-Jansen et al., 2018; Schreuders et al., 2018).

The TPJ/STS was frequently identified relating to adolescent prosocial decision-making (Van Hoorn et al., 2016; Sakai et al., 2017; Tousignant et al., 2018; Will et al., 2018) and prosocial choices (Tashjian et al., 2018; Do et al., 2019; Schreuders et al., 2019; Spaans et al., 2020). Young adults continued showing activation in the TPJ/STS (van den Bos et al., 2009; Telzer et al., 2011; Lemmers-Jansen et al., 2018; Schreuders et al., 2018; van der Meulen et al., 2016), and there was additional evidence that activation during prosocial tasks in this region increases with age bilaterally (van den Bos et al., 2011; Van Hoorn et al., 2016; Tousignant et al., 2018). The IFG bilaterally was less often related to prosocial cognition, but it was consistently identified with studies that also found activation in the TPJ (Sakai et al., 2017; Lemmers-Jansen et al., 2018; Schreuders et al., 2018; Will et al., 2018; Do et al., 2019; van der Meulen et al., 2016).

Lastly, Duell et al. (2021) found that DMN regions, including the PCC, TPJ, and MTG, had activation relating to prosocial decision-making that interacted with adolescent testosterone and cortisol levels.

#### Summary

Twenty-one studies found DMN regions related to prosocial cognition during adolescence, and several found evidence suggesting developmental increases in DMPFC and TPJ activation during prosocial tasks. In general, the mPFC and TPJ regions were commonly identified across both prosocial decision-making and prosocial choices, but other regions including the PCC, precuneus, and IFG may be significant in certain contexts.

### Salience Network Regions

In the reviewed literature, the salience network, consisting of the anterior cingulate cortex (ACC) and bilateral insula, had increased activation to prosocial decision-making relative to a calculation control (Sakai et al., 2017), had greater activation in prosocial decision-making after observing highly prosocial peers relative to low prosocial peers (Duell et al., 2021), and had greater activation in young adults compared to younger adolescents when making forgiveness decisions (Moor et al., 2012). Increased dorsal (d)ACC and bilateral insula activation also positively related to prosocial giving frequency when youth made *antisocial* choices, such that greater activation while behaving antisocial related to greater giving frequency in both adolescents (van den Bos et al., 2011) and young adults (van den Bos et al., 2009).

Activation in the dACC showed a relationship with overall giving frequency, although in one study this relationship was positive (Moor et al., 2012), while in another it was negative (Do et al., 2019). Studies also identified the ACC without the insula during prosocial decision-making (van den Bos et al., 2011; Lemmers-Jansen et al., 2018; Will et al., 2018), prosocial choices (Telzer et al., 2013), and prosocial choices when regressed with a prosocial questionnaire in young adults (Telzer et al., 2011). Prosocial inclination also related to greater cortical thinning in the dACC across adolescent development (Ferschmann et al., 2019) and greater resting state functional connectivity between the ACC and the bilateral PCC, middle cingulate cortex, and right precentral gyrus (Okada et al., 2019). This same study also used magnetic resonance spectroscopy and found that gamma-aminobutyric acid (GABA) was the only neurotransmitter in the ACC to relate to prosocial inclination (Okada et al., 2019).

Insula activation during social observation tasks positively related to prosocial behavior frequency following the task in both adolescence (Masten et al., 2010) and young adults (Tousignant et al., 2018), as well as prosocial decision-making across development (Tousignant et al., 2018). Interestingly, the right insula had greater activation while subjects behaved prosocially to friends more than disliked peers in early adolescence (Schreuders et al., 2019), but in young adults, right insula activation was negatively related to prosocial giving frequency to friends compared to disliked peers (Schreuders et al., 2018). Yet other studies still found that when young adults made prosocial choices, they had greater insula activation when distributing money (Güroğlu et al., 2014) and when playing Cyberball (van der Meulen et al., 2016).

#### Summary

Seventeen studies reviewed here found that the salience network was involved in prosocial behavior across adolescent development, but its exact relationship to enacting prosocial behavior remains unclear. Some studies found that salience network regions positively related to prosocial decisions, while others indicated the opposite. Overall, this network appears most related to prosocial cognition when accounting for the frequency of prosocial decisions across an entire task rather than activation specific to adolescent prosocial choices.

### Control Network Regions

The control network, consisting of the bilateral LPFC and inferior parietal lobule (IPL), related to prosociality in adolescence and more so in young adulthood. Right-lateralized DLPFC and IPL were related to prosocial decision-making compared to non-prosocial calculation in adolescents (Sakai et al., 2017), and the bilateral DLPFC and left IPL related to socially mindful decision-making in young adults, with the right DLPFC and left IPL specifically relating to prosocially mindful choices (Lemmers-Jansen et al., 2018). Both the left DLPFC and right inferior parietal sulcus related to greater cortical thinning in high prosocial compared to low prosocial adolescents (Ferschmann et al., 2019). While both adolescents and young adults showed activation in the bilateral IPL when making prosocial choices to friends more than disliked peers (Schreuders et al., 2018, 2019), only young adults showed increased activation in the right DLPFC to this contrast, and its activation negatively correlated with giving inequity that favored friends more than disliked peers (Schreuders et al., 2018). Similarly, young adults had greater activation in the left DLPFC and bilateral IPL when making prosocial choices, and they had activation in the left DLPFC that positively related to prosocial feelings toward one’s family (Telzer et al., 2011). The right IPL related to prosocial giving frequency in adolescents and young adults while playing the Dictator Game after viewing social scenes (Overgaauw et al., 2014). The right DLPFC related to decision-making across adolescents and young adults (Tousignant et al., 2018) and prosocial choices in young adults (Güroğlu et al., 2014), and one study found greater activation in young adults compared to adolescents during decision-making (van den Bos et al., 2011). The left DLPFC showed activation across adolescence in response to prosocial choices (Spaans et al., 2020), left DLPFC activation during prosocial choices changed across early to mid-adolescence (Do et al., 2019), and its activation during decision-making was modulated by testosterone and cortisol concentrations (Duell et al., 2021).

#### Summary

Thirteen studies found CN regions relating to prosocial decision-making, choices, and choice frequency. In general, these findings suggest that DLPFC activation is more pronounced in young adults, especially during choices, and most findings suggest that greater DLPFC activation is related to greater prosocial and equitable choices.

### Visual and Somatosensory Regions

Ten studies found that visual regions such as the middle occipital gyrus (Will et al., 2018; Schreuders et al., 2019; Duell et al., 2021), cuneus (Telzer et al., 2013; Lemmers-Jansen et al., 2018; van der Meulen et al., 2016; Duell et al., 2021), fusiform gyrus (Tousignant et al., 2018; Will et al., 2018; Duell et al., 2021), calcarine gyrus (Schreuders et al., 2018; Will et al., 2018), and visual cortex generally (van den Bos et al., 2009, 2011) were involved in both prosocial decision-making and choices across adolescence and young adults, and across tasks including the Dictator Game, the Trust Game, the Socially Mindful task, and Cyberball. One study also found that adults recruited more activation in the right fusiform face area than adolescents during prosocial decision-making while playing Cyberball (Tousignant et al., 2018).

Six studies found somatosensory regions related to prosocial behavior in the precentral gyri (Schreuders et al., 2018, 2019; Okada et al., 2019; Duell et al., 2021), the right postcentral gyrus (Sakai et al., 2017; Schreuders et al., 2019; Duell et al., 2021), and the supplementary motor area (Schreuders et al., 2018; Will et al., 2018). These findings were in both adolescents and young adults, and in studies focusing on prosocial decision-making, choices, frequency, and prosocial inclination from resting state functional connectivity.

#### Summary

Although not often hypothesized, both visual and somatosensory regions were implicated in eleven prosocial cognition studies from early adolescence to young adulthood and relating both to the prosocial decision-making process in general as well as making prosocial choices.

## Discussion

**FIGURE 2:**
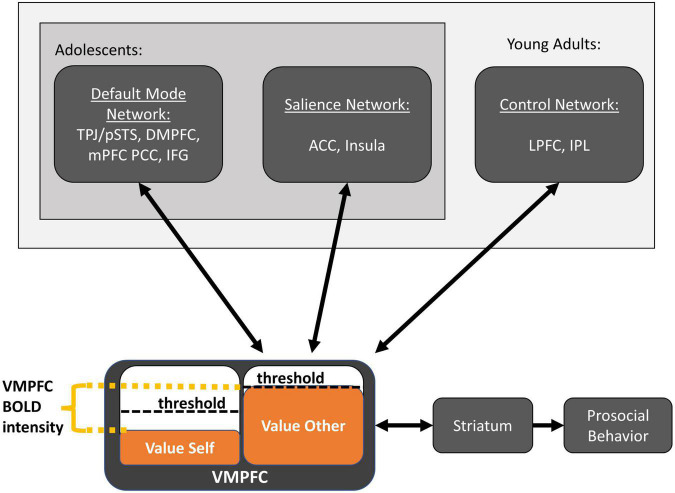
The domain-general developmental “Do-GooD” network model of prosocial cognition. Three domain-general networks contribute value computations for prosocial decisions, which are integrated in the ventromedial prefrontal cortex. Activation in the ventromedial prefrontal cortex and striatum corresponds to the relative value between available options. Once the prosocial option reaches the decision-threshold, prosocial behavior is enacted. The default mode network predominantly computes value for self and other, and these value computations can be influenced by events that occur before the prosocial decision and may reflect general prosocial disposition. The salience network may contribute at least two types of information, one is value attributed to affect while another is self-monitoring for fairness norms, the latter of which may suppress value accrual to uphold perceived fairness. The control network computes the value for abstract desires and learned social norms and contributes this value in ways that align with those desires and norms. The control network is involved across adolescence, but importantly, it further develops through young adulthood with greater activation that increases the weight of these social norm values during decision-making. This model proposes a bidirectional process between value computation and value accrual that unfolds over many cycles in time, and where response time is proportional to the decision threshold in the ventromedial prefrontal cortex.

In the following, we propose a synthesis of the above findings in terms of domain-general brain networks (Uddin et al., 2019). Our proposed Domain-General Developmental “Do-GooD” Network Model of Prosocial Cognition in Adolescence is shown schematically in Figure 2. Specifically, we propose that the general mechanism of prosociality in adolescents follows value-based decision-making, with three domain-general networks contributing computations as follows: the default mode network computes value predictions for both the self and other, the salience network assesses fairness to modulate value accrual, and the control network develops throughout adolescence to compute value for upholding social rules and norms. The contributions of these three networks are integrated in the VMPFC and striatum, as described below.

### Ventromedial Prefrontal Cortex and Striatum

Value-based decision-making is a framework for general decision-making in the brain, and it is thought to be especially suited for describing both altruism (Brosch and Sander, 2013) and adolescent development (Pfeifer and Berkman, 2018). This framework proposes that the VMPFC integrates value computations from other brain regions (Brosch and Sander, 2013). Prior work demonstrates that VMPFC activation reflects the relative subjective value of selected options (Boorman et al., 2009; Nicolle et al., 2012) together with the VS (Lim et al., 2011). Work by Juechems et al. (2019) helps disentangle the role of the striatum from the VMPFC, showing that the striatum encodes the outcome’s value receipt independent of goals, while the VMPFC encodes a “representation of cumulative assets in a way that maximizes a specific goal” (pg. 984). However, an exact understanding of the VMPFC and striatum’s roles in value-based decision making remains unclear.

Neurocomputational research may help provide further evidence for the differing roles of the VMPFC and VS during prosocial cognition. Hutcherson et al. (2015) developed a neurocomputational model that predicted altruistic decision-making in adults using activation in the VMPFC, VS, and TPJ with strikingly accurate results within and across subjects. In their model, behavior was executed once VMPFC activation representing the decision’s relative value exceeded a decision-threshold. TPJ activation accounted for computations regarding the value of giving to others whereas the VS had greater activation during selfish decisions, and the VMPFC responded to both selfish and prosocial decisions (Hutcherson et al., 2015). Other neurocomputational work on prosocial *learning* suggests that the VS and VMPFC activations were also related to prediction error (PE) signals in adolescents, where the VS represents PE while learning for oneself while the VMPFC represents PE while learning for others (Westhoff et al., 2021). However, in a previous adult study using the same experiment, PE in the VS was impartial to self-versus other, and the subgenual ACC contributed to prosocial PE (Lockwood et al., 2016). While the VMPFC, subgenual ACC, and VS are all anatomically close and may partially overlap, these discrepancies could also reflect subtle differences between adult and adolescent prosocial learning mechanisms related to value encoding. Overall, these results align well with and expand the value-based decision-making framework for prosocial behavior, with value accrual taking place in the VMPFC, value outcome in the striatum, and value computation in the TPJ (and possibly other regions/networks, as we discuss below).

Studies reviewed here supported and expanded on these findings into the adolescent age range. The VMPFC and VS had greater activation during prosocial behavior, and the VS had significant functional connectivity with both mentalizing (pSTS, medial PFC) and control regions (VLPFC) during prosocial decision-making (van den Bos et al., 2009; Telzer et al., 2011, 2013; Güroğlu et al., 2014; van der Meulen et al., 2016; Lemmers-Jansen et al., 2018; Tousignant et al., 2018; Do and Telzer, 2019; Spaans et al., 2020; Brandner et al., 2021; Duell et al., 2021). Furthermore, adolescent reaction times for making prosocial choices in the Dictator Game were longer than selfish choices (Will et al., 2016; Do et al., 2019), which aligns well with a bidirectional mechanism involving both value computation and accrual over time.

### Default Mode Network

The default mode network (DMN) subsumes the mentalizing regions of the brain (Lombardo et al., 2010; Hyatt et al., 2015). The DMN’s involvement in prediction (Dohmatob et al., 2017), social information consolidation (Meyer et al., 2019), and goal orientation (Spreng et al., 2014) makes it a likely candidate for a system that computes both self- and other-value. Most studies reviewed here identified key mentalizing and DMN nodes relating to prosocial behavior, and activation of DMN regions outside of direct prosocial decision-making also predicted subsequent prosocial behavior (Masten et al., 2010; Tashjian et al., 2018). Evidence suggests that some DMN regions have specificity for self-processing, such as the PCC and mPFC (Blakemore et al., 2007; van Schie et al., 2018), with others being more specific for other-processing, such as the DMPFC and TPJ (Mason and Just, 2009; Sabbagh et al., 2009; Carter and Huettel, 2013). However, these associations are not always the case and vary based on context (Nicolle et al., 2012) and subregional functionalization (Molnar-Szakacs and Uddin, 2013). We propose instead that the computation for self- and other-value occurs throughout the DMN and that the resulting information accumulates in the VMPFC for value integration. It should be noted that the VMPFC is also considered to be a key DMN region (Uddin et al., 2019), so while we differentiate our discussion of the VMPFC from the DMN for clarity, it is likely meaningful that a value accrual mechanism is integrated with the value computation mechanisms so postulated.

The mirror neuron system may also act as a neural substrate upon which mentalizing regions simulate others (Gallese and Goldman, 1998), and thus may be relevant to computing other-value. The mirror neuron system consists of some DMN regions, such as the IFG and STS, as well as other regions, including the primary motor and occipital cortices (Rajmohan and Mohandas, 2007). The mirror-neuron system involvement helps explain why regions such as the motor cortex (Sakai et al., 2017; Okada et al., 2019; Schreuders et al., 2019; Duell et al., 2021) and occipital cortex (van den Bos et al., 2009, 2011; Telzer et al., 2011, 2013; van der Meulen et al., 2016; Lemmers-Jansen et al., 2018; Duell et al., 2021) had activation relating to prosocial behavior. Supporting this relationship between mirroring and prosocial decision-making, the electroencephalography (EEG) signal from adult subject’s dorsal somatosensory cortex while observing another’s hand being struck (by a belt) accounted for the amount of money donated to reduce subsequent strike intensity, and transcranial magnetic stimulation (TMS) disruption of the same region accordingly impaired that prosocial giving (Gallo et al., 2018). The mirror neuron system has a rich functional relationship with the DMN that has been shown to contribute to embodied simulation for both self and other processing (Lombardo et al., 2010), constituting a likely module leveraged by core DMN regions to compute self- and other-value.

### Salience Network

While the DMN plays a significant role in computing the value for both self and other, it needs not be the only system that contributes value. The salience network (SN) may also contribute to value computations in emotional contexts such as giving to friends (Schreuders et al., 2019), to family (Telzer et al., 2013), to previous exclusion (Moor et al., 2012; van der Meulen et al., 2016), or self-chosen charities (Spaans et al., 2020; Duell et al., 2021). The reviewed literature and other research also highlight another special involvement of the SN in prosocial decision-making: it appears to assess *fairness imbalance* to modulate value accrual.

Most neuroimaging studies on adolescent altruistic prosociality implement the Dictator Game or one of its many variations. The key element is that “Dictator” decides how to distribute a resource (usually money) between two people, generally themselves and/or some other person/player(s). Prosocial behavior can be operationalized in terms of the *fairness* in the resource distribution, where the distribution can favor the Dictator (selfish choices), the other player (prosocial inequity), or both evenly (prosocial equity). Prior work on the Dictator Game has shown that the amount of money that a Dictator keeps and gives was based on the *entire experimental session* according to what that Dictator determined to be a “fair” distribution, even if that Dictator played with a new recipient each trial (Bolton et al., 1998). Thus, determining fairness across all trials is an integral part of prosocial decision-making in this context.

The SN consists primarily of the ACC and insula (Uddin et al., 2019), and a meta-analysis found the SN regularly active when processing unfair offers (Gabay et al., 2014). In a study with ages ranging from 10- to 20-years-old, all ages showed dACC and bilateral insula activation when processing unfairness, and specifically when the adolescent themselves deviated from their own sense of fairness (Güroğlu et al., 2011). That is, the SN was self-monitoring unfairness in their own behavior, and activation in the SN was strongest to personal-norm fairness violations in those adolescents who mostly behaved fairly (Güroğlu et al., 2011). Therefore, strong activation in the SN during prosocial decision-making suggests an experience that one’s actions are not aligned with their personal sense of fairness. The ACC in particular has also been shown to have significant functional connectivity with the VMPFC to provide information about task switching that optimizes long-term payoff (Economides et al., 2014). This could be especially relevant to fairness considerations, as a signal that payoffs are unfair demands switching one’s strategy in subsequent trials to optimize fairness. This may additionally indicate that VMPFC and ACC connectivity is an important source of feedback both within and across prosocial task trials.

Some of the reviewed studies on adolescent prosocial behavior found the dACC positively related to giving (van den Bos et al., 2009, 2011; Moor et al., 2012; Duell et al., 2021), yet another found the dACC negatively related to giving (Do et al., 2019); however, considering the dACC as encoding personal unfairness can resolve this discrepancy. van den Bos et al. (2009, 2011) found that dACC activation while choosing to act Selfish > Prosocial related to greater overall giving, suggesting that processing selfish behavior as unfair conferred greater prosocial behavior throughout the experimental session. Similarly, Do et al. (2019) found that dACC activation during the prosocial choice contrast Costly Giving > Costly Reward negatively related to overall prosocial giving; that is, feeling that costly giving was unfair led to decreases in overall giving. Moor et al. (2012) found that dACC activation during decision-making positively related to more “forgiving” offers to players who had previously Excluded > Included them. Because the adolescents were previously excluded, most offers were highly unequal and punishing, but those who had greater dACC activation possibly found that punishment overly unfair and subsequently gave more prosocial offers. Lastly, Duell et al. (2021) found the dACC had increased activation when behaving prosocially after having just observed a highly prosocial peer donate to charity. Observing a highly prosocial peer may have increased fairness self-monitoring in subsequent giving that increased dACC activation and conferred more prosocial decisions. In some of these studies, activation in the dACC during either prosocial or selfish contrasts could predict overall prosocial behavior. Fairness considerations are based on the entire experimental session, not trial-by-trial, so it could be that the dACC modulates the ongoing value accrual in the VMPFC to suppress unfair options. Supporting this possible mechanism, Okada et al. (2019) found that the only neurotransmitter in the ACC that related to a prosocial questionnaire was GABA, responsible for neuronal inhibition.

### Control Network

The control network (CN) consists of the LPFC and the IPL (Uddin et al., 2019). The LPFC structurally and functionally develops during adolescence (Sowell and Jernigan, 1998; Dumontheil, 2014), which suggests its role in prosocial decisions may also develop during this time. The LPFC represents abstract and multi-dimensional values (Dixon and Christoff, 2014), such as delayed-discounting (Guo and Feng, 2015), social norms (Hackel et al., 2020), and moral attitudes (Crockett et al., 2017). Prosocial decision-making is frequently multi-dimensional, it includes social other recognition, understanding goals related to context specific social norms, and the cost-benefit-analysis for different options. The CN within a value-based decision-making framework may contribute value reflecting abstract goals and social rules rather than strictly suppressing selfishness (Berkman et al., 2017). Thus, the CN develops to contribute value that accounts for these contextual goals and learned moral norms in the decision-making process to support prosocial decisions.

In the reviewed literature, the DLPFC and IPL were frequently engaged in prosocial decision-making and choices, and the DLPFC specifically showed evidence for development across adolescence relating to prosocial behavior, both in brain structure (Ferschmann et al., 2019) and function (van den Bos et al., 2011; Do et al., 2019). Interestingly, the right DLPFC showed activation in young adults when the recipient of their prosocial behavior was unknown and not depicted (van den Bos et al., 2011; Güroğlu et al., 2014; Lemmers-Jansen et al., 2018; Tousignant et al., 2018), whereas the left DLPFC showed activation in young adults when giving prosocially to their families (Telzer et al., 2011), to a visible other (Do et al., 2019), or to a charity of their choosing (Spaans et al., 2020; Duell et al., 2021). The DLPFC accounting for abstract social values also resolves a discrepancy between most studies finding greater activation to prosocial choices, but Schreuders et al. (2018) finding that greater activation during decision-making to friends compared to disliked peers *negatively* related to giving inequity that favored friends more than disliked peers. It could be that giving equally to disliked peers requires more value for abstract social norms than does giving only to close friends, and thus giving to a disliked peer would necessitate greater recruitment in regions that account for this abstract virtue of “giving despite disliking the peer.” The IPL overall had less frequent activation related to prosocial behavior; it was bilaterally active when mid-adolescents gave money to Friends > Unknown peers (Schreuders et al., 2019) and when young adults gave to their family compared to receiving a reward (Telzer et al., 2011). While evidence suggests that the CN was more involved in older adolescents and young adults, it is unclear what exactly this network contributes to prosocial neural mechanisms; however, because previous work shows that the CN represents abstract values, its involvement could be illuminated through individual differences in social values and behavior.

Most individual differences in brain activation in response to social norms were demonstrated in the CN, DMN, and reward regions. When adolescents decided to give prosocially to their family, those with a greater sense of family obligation had more activation in the striatum (Telzer et al., 2013), greater activation in the right TPJ, bilateral pSTS, right ACC, and left DLPFC, as well as greater functional connectivity between the VS and the left VLPFC, left DMPFC, and right mPFC (Telzer et al., 2011). Culture likely plays a role, as evidenced by a study finding that adolescents from a communalist culture had greater striatum activation when giving to their family than those from an individualist culture (Telzer et al., 2010). The LPFC contributes value regarding social rules and expectations, especially when they are different from one’s disposition. In adults, greater right DLPFC connectivity to the VMPFC occurred when abiding by prosocial or selfish norms different from the individual’s prosocial disposition (Hackel et al., 2020). Furthermore, transcranial magnetic stimulation of the DLPFC in adults increased prosociality (Balconi and Canavesio, 2014). Prosocial giving toward in-groups versus out-groups also shows individual differences, where functional connectivity between the VS and pSTS can either indicate greater bias or lesser bias depending on which group the individuals are giving when the functional connectivity is greatest (Do and Telzer, 2019). Yet not many studies investigating adolescent prosocial behavior manipulate social norms or relate brain activation to cultural values, both of which may better elucidate how neural correlates relate to individual perceptions of social norms and how one could leverage this relationship to increase prosociality.

### Limitations

This review has several limitations. First, substantial study heterogeneity precluded a meta-analysis of this literature. Such a meta-analysis would greatly assist in the interpretation of this work, including the discussion of the TPJ, which has unclear and disputed anatomical boundaries (Carter and Huettel, 2013; Geng and Vossel, 2013; Schurz et al., 2014). However, meta-analyses cannot currently accommodate functional connectivity or magnetic resonance spectroscopy findings, both of which contribute to the current review and model proposed here. Second, while our model proposes a domain-general network framework, there are not enough connectivity studies to make strong claims about the networks as a whole, and not about which brain regions definitively constitute those networks. Given this limitation, our aim is to present a network-informed model aligned with the current evidence to drive brain connectivity hypotheses for future work on prosocial development. Third, most studies reviewed here investigated prosocial neural correlates during a task that involved sharing or keeping money, namely the Dictator Game (or a variation). While monetary giving may be a convenient operationalization for prosocial decision-making, it limits the scope and generalizability of these findings, as it may be only one dimension of prosocial behavior in adolescent lives. Evidence also shows that introducing monetary transactions during social decisions can change the social context of that situation (Gneezy and Rustichini, 2000; Mellström and Johannesson, 2008). Indeed, while our model suggests the role of fairness detection in prosocial decision-making, this may be specific to economic prosocial contexts rather than prosociality in general. An important direction for new research would be to compare prosocial neural correlates in situations that involve resource distribution with neural correlates observed in situations that, for example, involve prosocial emotional consolation.

### Future Directions

#### Methodological Advances

Future studies could benefit from new methodological approaches to study neural correlates of prosociality. As previously mentioned, prosocial paradigms thus far use games in which adolescents distribute a resource (usually monetary) between themselves and others. However, everyday life includes prosocial behavior that does not incorporate resource distribution, such as empathetic listening, playing cooperative games, or expressing love and kindness for friends and family. Future studies on prosocial behavior could adapt this real-life game observation during moments of cooperation or selfishness to investigate questions about prosocial cognition in ecologically valid contexts for adolescents. On the MRI methodological side, connectivity studies have been sparse. The reviewed literature on prosocial cognition implicates many brain regions, yet it remains unclear how these regions coactivate in networks during tasks to support prosocial behavior. Furthermore, only one study we reviewed had considered resting state functional connectivity, which may be especially useful to understand individual differences in prosocial disposition. The brain’s structural connectivity may also offer insights into prosocial disposition and one’s change in prosociality with development.

#### Model Predictions

The “Do-GooD” model predicts that greater activation of key other-value computing regions (e.g., the TPJ) through TMS could increase prosocial giving, as was similarly demonstrated in the DLPFC, which also resulted in increased prosociality (Balconi and Canavesio, 2014). With respect to developmental changes, we expect both prosociality and its associated brain network connectivity to increase during adolescent development. Specifically, we expect prosociality to increase in tandem with functional within- and between-network connectivity development in the DMN, SN, and CN, which has previously been shown in Ryali et al. (2016), and which may be associated with changes in myelination. Furthermore, because studies find that resting state functional connectivity shows relationships similar to those during tasks (Smith et al., 2009), it may be fruitful to test whether individual prosocial tendencies are associated with differences in resting state connectivity. The proposed model could also help develop new targets for interventions and for monitoring changes. Specifically, we may expect that interventions aimed at increasing prosociality, such as love and kindness meditation, will increase functional and structural connectivity within the DMN and between it and the mirror neuron system, thus improving the computation of other-value. Furthermore, interventions that promote prosociality as an intrinsically good or “fair” option, which would *decrease* SN activation while giving and *increase* SN activation while behaving selfishly, each correlating with the intervention’s effectiveness. SN activation toward prosocial versus selfish decisions as well as functional connectivity between the DMN, mirror neuron system, and CN all could be used to assess adolescents with challenges relating to low prosocial behavior, such as conduct disorder or the early development of a potential antisocial personality disorder, and inform treatment planning. For example, the intervention could emphasize communal responsibility in those with strong SN activation to the (un)fairness of prosocial options or instead emphasize human shared experiences to enhance other-value computations between the DMN and mirror neuron system.

## Conclusion

In this review, we summarize and synthesize the current neuroimaging findings on prosocial behavior during adolescence. We propose that prosocial decision-making is a form of value-based choice carried out by domain-general networks. In particular, we suggest that overall value accrues in the ventromedial prefrontal cortex and reward regions, value for self and others is computed in the default mode network, fairness imbalance is monitored in the salience network, and abstract values for social norms develops during adolescence in the control network. Ultimately, understanding the neural basis and development of prosocial behavior is crucial to understanding how cooperation can be promoted in a society. This neuroscientific understanding may help illuminate the underlying neural development of psychiatric disorders, such as conduct disorder or antisocial personality disorder, and aid in the development and evaluation of improved and innovative treatments for these conditions.

## Data Availability Statement

The original contributions presented in the study are included in the article/supplementary material, further inquiries can be directed to the corresponding author/s.

## Author Contributions

BS did the conceptualization, performed the methodology, carried out the formal analysis and data curation, investigated the data, wrote the original draft, reviewed, and edited the manuscript. TY did the conceptualization, performed the methodology, wrote, reviewed, and edited the manuscript, and carried out the funding acquisition. KP and NJ performed the methodology, and wrote, reviewed, and edited the manuscript. OT did the conceptualization, performed the methodology, wrote, reviewed, and edited the manuscript, and carried out the project administration and funding acquisition. All authors contributed to the article and approved the submitted version.

## Conflict of Interest

The authors declare that the research was conducted in the absence of any commercial or financial relationships that could be construed as a potential conflict of interest.

## Publisher’s Note

All claims expressed in this article are solely those of the authors and do not necessarily represent those of their affiliated organizations, or those of the publisher, the editors and the reviewers. Any product that may be evaluated in this article, or claim that may be made by its manufacturer, is not guaranteed or endorsed by the publisher.
